# Perception of team conflict and goal commitment over time: a multilevel analysis in sports teams

**DOI:** 10.3389/fpsyg.2026.1935278

**Published:** 2026-10-28

**Authors:** Jonas Lüdemann, Jens Kleinert

**Affiliations:** 1 Section Health & Social Psychology, Institute of Psychology, German Sport University Cologne, Cologne, Germany; 2 German Research Centre of Elite Sport (momentum), German Sport University Cologne, Cologne, Germany

**Keywords:** Bayesian estimation, commitment, group dynamics, intragroup conflict, sport, team, team conflict, team goal

## Abstract

**Objective:**

Despite evidence showing how team conflict affects performance, the factors influencing conflict remain largely unclear. One possible factor influencing team conflict is how goals are handled. When dealing with team goals, team goal commitment is an important variable. Team goal commitment describes how connected team members feel to the team’s goal and how determined they are to achieve it. The objective of this study was to examine whether team goal commitment predicts team conflict in sports teams. Method: Soccer teams were surveyed using a two-wave design. The sample comprised 356 players (123 female, 232 male, one not specified; *M*_*a*ge_ = 25.14, *SD*_age_ = 5.03) from 25 teams. Team goal commitment (both personal commitment and perceived commitment of other team members) was measured with the goal commitment scale adapted to team goals. Team conflicts were measured using the intragroup conflict scale for sport. Analyses were performed in R using Bayesian multivariate multilevel regression and response surface methodology.

**Results:**

Initial perceptions of conflict positively predicted levels of conflict 2 months later. Before the initial level of conflict was included in the analysis, a negative relation appeared to be found between perceived team goal commitment of the other team members and process conflict 2 months later. However, this association disappeared once the baseline conflict level has been controlled for. Relationship and process conflict were less prevalent in women’s teams than in men’s teams.

**Conclusion:**

Perceived high levels of conflict in teams should be addressed early on, as there is a high chance that they will remain otherwise. Future studies should examine the interaction between team goal commitment and process conflict more closely.

## 1 Introduction

Team collaboration can always involve conflicts (De Dreu and Weingart, 2003). Over the past three decades, team conflict and its effects have been extensively studied, revealing their impact on performance, well-being, and group development in various environments, including work and sports teams (De Dreu, 2026; Secaras et al., 2026). While there is much empirical evidence on the types of conflict and their effects, there is comparatively little evidence on how team conflict emerges and evolves over time (Korsgaard et al., 2014; Shah et al., 2021; Cronin and Bezrukova, 2019). Thus, furthern investigations of team conflict over time seem necessary, both for a comprehensive scientific explanation and for the management of team conflict in practice (e.g., in work projects or sports; De Dreu, 2008; Lüdemann and Kleinert, 2025; DeChurch et al., 2013).

The emergence of team conflicts has various sources, including, among others, contradictory goals, power imbalances, diversity, and cultural values (Korsgaard et al., 2014). Of these sources, goals are a particularly promising source of team conflict to examine because goals are central to both collaboration (e.g., group flow; Pels and Kleinert, 2023) and non-collaboration (e.g., team conflict; Nesterkin et al., 2016) in teams. Thus, goals can always be an indicator of conflict potential within the team (Tjosvold et al., 2014). That is, the potential for conflict tends to be lower when the goals within the team are largely compatible with each other, and the conflict potential tends to be higher when the goals are meaningfully contradictory. Furthermore, previous research has shown that working with goals is widely used in practice and is an effective method for collaborative performance (Kleingeld et al., 2011; Forsblom et al., 2019), thus reducing the potential for team conflict. Therefore, the objective of this study is to investigate how team conflict can be explained by goals in soccer teams over time.

Team conflict is defined as a dynamic process that occurs between or among interdependent parties when they experience incompatibility or interference in the achievement of their goals or interests (Korsgaard et al., 2008). Previous research on team conflict distinguishes between relationship, task, and process conflict (Jehn, 2014). Relationship conflict refers to interpersonal incompatibilities among team members regarding different norms, personalities, and values. Task conflict refers to disagreements among team members about the content and outcome of the common task, while process conflict refers to disagreements among team members about strategies for accomplishing the task (De Wit et al., 2012; Jehn and Bendersky, 2003).

In relation to the objective of this study, team conflict may arise from team goals under two conditions. On the one hand, team goals can lead to team conflict if the team goal is perceived as incompatible with the personal goal (goal incompatibility; Korsgaard et al., 2008; Lewin, 1948). On the other hand, team goals can lead to team conflict if team members do not agree on the value or importance of the team goal (goal importance; Fitzsimons et al., 2016). Both conditions are associated with a low team goal commitment.

Team goal commitment in general means that team members feel connected to the team goal and are determined to achieve it (Aubé and Rousseau, 2005; Weldon and Weingart, 1993). According to the processes described above, team goal commitment depends on both, the amount of goal compatibility and the amount of goal importance. This means, if the team goal is incompatible with personal goals, or if the team goal is perceived as valueless, team members may feel little connection to the team goal and little determination to achieve it.

Moreover, team goal commitment depends on the constellation between the individual’s own team goal commitment and the individual’s perception of the team goal commitment of the other team members (Weldon and Weingart, 1993). That means, individuals not only experience how strongly they feel connected to the team goal and how determined they are to achieve it (personal goal importance), but they also perceive and can be influenced by how strongly other team members feel connected to the team goal and how determined those other team members are to achieve it (goal importance of the others). If there is an incongruence between the personal goal importance and the perceived goal importance of the other team members, this may have an impact on team goal commitment and be associated with a higher potential for team conflict. The potential for team conflict is particularly high when the personal goal importance is high and the individual’s perception of the goal importance of the other team members is low, because this constellation threatens the strong personal connection to the team goal and the personal initiative to achieve it. In contrast, if the personal goal importance is low and the goal importance of the other team members is high, the potential for team conflict is rather moderate, because there is no threat to personal connection or initiative to the team goal. Moreover, if the personal goal importance and the goal importance of the other team members are congruent, that is, both low or both high, no further effect on team goal commitment is expected, and consequently, no further effect on team conflict.

In current research, there is little evidence about the relation between team conflict and team goal commitment, and it is not entirely consistent. Consistent with the theoretical assumptions, previous research has identified two particular indications regarding the relationship between team conflict and team goal commitment. First, indications for moderate negative correlations between team conflict and team goal commitment were shown. Second, indications for moderate positive correlations were found between team conflict and incongruences among team members’ personal team goal commitment. However, the findings were not entirely consistent, as the indications for negative correlations between team conflict and team goal commitment were not always confirmed.

More specifically, the associations between team conflict and team goal commitment have been addressed in four empirical publications so far. In these publications, the relationship between team conflict or conflict-related constructs and team goal commitment was investigated in student teams or work teams. In particular, Park et al. (2019) found negative associations between team conflict and personal team goal commitment among members of student project groups. Consistently, van Mierlo and Kleingeld (2010) found negative relations between intra-team competition (as conflict-related construct) and personal team goal commitment in an experiment with teams of undergraduate students performing an interdependent task (in this task, the participants worked in groups of three and had to build a tower of blocks together; van Mierlo and Kleingeld, 2010). However, it should be noted that the result was not found in the first run of the experiment (van Mierlo and Kleingeld, 2010). In the working context, two empirical studies have so far provided evidence in support of the theoretical assumptions. First, Aubé and Rousseau (2011) examined interpersonal aggression (as conflict-related construct) and team goal commitment in work teams at the team level, and reported negative associations between the two constructs. Second, in line with theoretical assumptions regarding the interaction between team members’ relative team goal commitment, Manata et al. (2021) examined interpersonal differences in personal team goal commitment within business project teams and found positive associations with problematic communication behaviors (as a conflict-related construct).

Besides research on the relationship between team conflict and team goal commitment, few empirical studies shed light on the relationship between team conflict and incompatibility between team and personal goals. Consistent with the theoretical assumptions, two experimental studies have shown that incompatibility between personal and team goals stimulates competitive individual strategies within an interdependent task (compared to conditions with no specific goal or with only a team goal in a letter-word-sentence game, Crown, 2007; Crown and Rosse, 1995).

In summary, the impact of team goal commitment on team conflict has not been extensively researched, resulting in a multidimensional research gap. First, no study has examined the impact of team goal commitment on team conflict longitudinally, so no predictive evidence is available. Second, no study has specifically examined the effect of incompatibility of team goals with personal goals on team conflict. Third, there is no study that has specifically examined the influence of an incongruent constellation between personal team goal commitment and the perceived team goal commitment of the other team members on team conflict. Fourth, no study has examined the impact of team goal commitment on team conflict in the team sport context.

Based on the theoretical assumptions and research gaps described above, the aim of this study was to examine whether and how team goal commitment can longitudinally predict team conflict potential in the team sport context. In general, team goal commitment is expected to negatively predict team conflict. Specifically, it is assumed that personal team goal commitment and the perceived goal commitment of the other team members at the beginning of the season negatively predicts team conflict 2 months later (Hypothesis 1). Furthermore, it is hypothesized that goal incompatibility between the personal goal and the team goal (as a possible explanation for team goal commitment) at the beginning of the season predicts team conflict 2 months later (Hypothesis 2). Finally, it is hypothesized that the constellation of the personal goal commitment and the perceived goal commitment of the other team members at the beginning of the season explains the level of team conflict 2 months later (Hypothesis 3). More specifically, it is assumed that a congruent constellation, that is, personal goal commitment and goal commitment of the others both low or, conversely, both high, is negatively related to team conflict. Accordingly, it is hypothesized that an incongruent constellation (i.e., high personal goal commitment and low goal commitment of the others, and vice versa) is positively related to team conflict.

## 2 Method

### 2.1 Sample

The study included soccer players aged 18 years and over whose teams played in low- to medium-high level amateur leagues. The convenience sample consisted of soccer players from 25 amateur teams (9 women’s teams, 16 men’s teams). At the beginning of the season (Time 1), a total of *N* = 356 adult soccer players (123 female, 232 male, one not specified; *M*_age_ = 25.14 years, *SD* = 5.03) were recruited. Two months later (Time 2), *n* = 173 players (63 female, 109 male, one not specified; *M*_age_ = 25.00 years, *SD* = 4.99) from the original sample were examined again. On average, the players at T1 had a mean team tenure of 3.16 years (*SD* = 3.38; range = 0–25 years). A personal goal for the season was reported by 89.9% of the players at T1. In addition, 69.7% of players reported that their team had a specific team goal, while 25.3% of the players reported that they were unsure about a specific team goal, but believed that there actually was one and 5.1% that that their team had no specific team goal.

Other demographic characteristics, such as socioeconomic status, sexual identity, racial identity, ethnicity, nativity, and immigration history, were not collected because they were not part of our theoretical assumptions. In the interest of a lean data collection process that avoids unnecessary data, we did not ask about these characteristics because we did not intend to draw conclusions based on them. However, to better understand adult amateur soccer in the study area, it can be viewed as a context in which people of different socioeconomic statuses and ethnicities come together, especially in metropolitan areas such as the one used in this study (Kearney, 2007).

### 2.2 Instrument

#### 2.2.1 Team goal commitment

To assess team goal commitment, the goal commitment scale by Klein et al. (2001) was adapted to team goals. Participants were asked to estimate their commitment to the team goal on a five-item scale (e.g., “I am strongly committed to pursuing our team goal”). All items were rated on a five-point response scale ranging from 1 (strongly disagree) to 5 (strongly agree). Higher scores represent higher personal team goal commitment. The internal consistency of the scale was acceptable, with Cronbach’s α = 0.75.

To assess the team goal commitment of other team members, again the commitment scale by Klein et al. (2001) was used. Participants were asked to rate how the five items of the scale applied to their team members (e.g., “My team members are strongly committed to pursuing our team goal”). All items were rated on a five-point response scale ranging from 1 (applies to none) to 5 (applies to all). Higher scores represent higher other team goal commitment. The internal consistency of the scale was acceptable, with Cronbach’s α = 0.78.

#### 2.2.2 Goal compatibility

A single item was used to measure the compatibility between the personal goal of the player and the respective team goal (“How much do your personal goal and the team goal match? Or do they even contradict each other?”). The item was rated on a 14-point response scale ranging from the poles “The goals contradict each other” (1) to “The goals match” (14). Higher scores represent higher perceived compatibility between the personal goal and the team goal, vice versa lower scores represent higher perceived incompatibility between the personal goal and the team goal.

#### 2.2.3 Team conflict

The Intragroup Conflict Scale for Sport (ICS-S) by Boulter et al. (2022) was used to measure team conflict. The ICS-S is a sport context-adapted version of the Behfar et al. (2011) intragroup conflict scale. The ICS-S consists of 11 items and comprises the subscales relationship conflict (RC, 4 items, e.g., “How much tension is there among members in your team?”), task conflict (TC, 3 items, e.g., “How frequently do members of your team engage in debate about different opinions or ideas?”) and process conflict (PC, 4 items, e.g., “To what extent do you disagree about the way to do things in your team?”). The items are assessed using a 9-point response scale from 1 (none/never) to 9 (very much/always). Higher scores represent higher perceived intensity of team conflict. Confirmatory factor analysis of the early season data at T1 supported the three-factor structure and showed good model fit: 𝛘^2^ (41) = 46.35; *p* = 0.261; RMSEA = 0.018; CFI = 0.998; SRMR = 0.050. Further, the internal consistency of the scales was acceptable, with Cronbach’s α = 0.87 for relationship conflict, Cronbach’s α = 0.76 for task conflict, and Cronbach’s α = 0.82 for process conflict.

### 2.3 Design and procedure

A nonexperimental longitudinal two-wave design was used. To obtain the convenience sample, 131 women’s and men’s soccer teams in the metropolitan study area were contacted in August 2024 before the start of the next season. A selective search was conducted for soccer teams in the relevant performance categories within the regional football association’s area of responsibility. Additionally, the study team’s existing networks were used to identify suitable teams. The respective contact persons, who were usually the coaches of the teams (otherwise also the club contact person or team captain), were contacted by telephone or email. Of the requested teams, 19.08% (25 teams) participated in the survey.

The surveys were conducted in the locker room before a training session. Study assistants informed the teams on site about the purpose of the study and then instructed them to complete the paper-and-pencil questionnaire. The players were instructed to complete the questionnaire without interacting with others. Study assistants were available to answer questions during the entire 10- to 15-min completion phase. The first survey (T1) took place at the start of the season at the beginning of September 2024 (between September 1, 2024 and September 15, 2024 or between the second and fourth matchday), and the second survey (T2) 2 months later (October 20, 2024–November 17, 2024 or between the ninth and twelfth matchday). This time interval was chosen because at least one-third of the championship had been completed by that point. This allowed the teams to assess their initial standing in the competition. At the same time, there were theoretically still enough opportunities for the teams to achieve their goals, even if major changes or efforts would have been necessary. To protect confidentiality, the questionnaires were matched over time using a coding system for each player.

All participants took part in the study voluntarily and gave written informed consent. The subjects were assured that all data would be treated strictly confidentially and anonymously, that participation in this study was voluntary, and that they could withdraw at any time during the survey without any consequences. Each team received €20 for participating. Additionally, the coaches were sent a summary of their team’s results, from which no conclusions could be drawn about individual team members.

The study was approved by the university’s ethics committee. Throughout the survey period, the study administrators were available to answer questions. Ultimately, only one coach reached out and asked for the summary of results again.

### 2.4 Statistical analysis

Data were analyzed using RStudio (R Core Team, 2025; Posit team, 2026). To examine how team goal commitment, goal compatibility, and the team goal commitment of the other team members at the start of the season predict the three types of team conflict RC, TC, and PC two months later, Bayesian multivariate multilevel regression models (Hox et al., 2018) via the brms package (Bürkner, 2017; Carpenter et al., 2017) were used (Hypothesizes 1 and 2). Additionally, multilevel polynomial regression (PR) and response surface methodology (RSM) in combination were carried out following recent methodological developments in group research (Berg et al., 2023) and Bayesian analysis (Hu, 2025; Hu and Ram, 2025) in order to examine how the constellation of the personal goal commitment and the others’ goal commitment at the beginning of the season are associated with the level of team conflict 2 months later (Hypothesis 3). The data in this study has three levels. The first level of data consists of individuals’ scores at the two measurement points. On the second level, the values are nested within the persons. On the third level, people are nested within the teams. The Bayes *R*^2^ and leave-one-out information criterion (LOOIC) were used to evaluate model fit. In model selection, lower values of LOOIC indicate better model fit (Bürkner, 2026).

#### 2.4.1 Bayesian multivariate multilevel regression models

The Bayesian estimation approach is a valid option for multilevel modelling, particularly when dealing with small sample sizes at the higher level. Furthermore, unlike Maximum Likelihood estimation, it can be shown that, under regularity conditions, the method converges to the posterior distribution if a sufficient number of Markov chain Monte Carlo (MCMC) iterations are used. In addition, uncertainty is derived directly from the posterior distribution; therefore, inference does not rely on normality assumptions when estimating variances (Hox et al., 2018).

Before computing the multivariate multilevel models, we applied reflection and inverse transformation to goal compatibility and team goal commitment, and reflection and log transformation to team goal commitment of others, to reduce their heavy left-skewness (Tabachnick and Fidell, 2014). This was done because our models assume linear associations with the outcomes, which can be better approximated with the transformed predictors. In addition, the transformations reduce the leverage of extreme observations, improve posterior geometry, and thereby increase Hamiltonian Monte Carlo (HMC) sampling efficiency. After transforming the predictors, they were group-mean centered for interpretation at the individual level, as our hypothesizes are about relationships between variables at the individual level (Hox et al., 2018; Enders and Tofighi, 2007). In addition, to account for right skewness in the three outcome variables, the multivariate multilevel models were estimated using a Student-t likelihood, which also increases robustness to residual outliers. Moreover, to handle missing values in the multivariate response variables, we performed imputation during model fitting in *brms*, thereby keeping the hierarchical and multivariate model structure (Bürkner, 2025). Specifically, missing values of RC, TC, and PC after 2 months were estimated conditional on all predictors included in the models under a missing-at-random assumption. Regarding the prior specification, we followed the recommendations of Depaoli and van de Schoot (2017) and derived prior estimates from a pilot study conducted with the same population. We used these estimates to specify weakly to moderately informative priors, which can be found in Supplemental material A.

Finally, two separate models were specified and estimated. Model 1 was performed to test hypotheses 1 and 2. To this end, we considered the transformed and group-mean-centered theoretical predictors of goal compatibility, team goal commitment, and team goal commitment of others at the start of the season. Additionally, we included age (group-mean centered) and gender (dummy coded) as control variables in the model. The three outcomes (RC, TC, and PC at 2 months later) were included in their original scale. Model 2 was an extension of Model 1 and additionally includes the (group-centered) baseline conflict levels for RC, TC, and PC at the beginning of the season.

All models were estimated Bayesian inference via HMC sampling. We ran six chains of 75,000 warmup iterations and 50,000 (Model 1) respectively 55,000 (Model 2) post-warmup iterations per chain, yielding 300,000 (Model 1) respectively 330,000 (Model 2) posterior samples in total. Following this, we conducted a series of diagnostic checks. We required a $\hat{R}$ value of ≤1.01, an effective sample size of ≥600 for all parameters, and a BFMI of ≥0.2 for all chains (cf. Baribault and Collins, 2025). Additionally, no divergences should be observed. Only iterations from models that met these criteria were used for inference.

#### 2.4.2 Multilevel polynomial regression and response surface methodology

The combined application of multilevel polynomial regression (PR) and response surface methodology (RSM) enables to examine (in)congruence hypothesis, that is, if the (in)congruence of two predictors (i.e., team goal commitment and team goal commitment of others) can explain a third depended variable (i.e., conflict type). The core idea of the method is to consider both predictors together while preserving their conceptual integrity, rather than combining the two different measures into a single value (i.e., difference scores; Edwards, 2001, 2002). In polynomial regression, in addition to the main effects, their product and the squared values of the main effects are also taken into account, which makes it possible to capture more complex interactions in the data. Subsequently, the response surface method is utilized as a form of interpretation of polynomial regression; it is not employed as a separate estimation method (Shanock et al., 2010). Rather, it assists in representing and interpreting the theoretical assumptions of (in)congruence in three-dimensional surfaces. Instead of interpreting individual regression coefficients, the resulting 3D surface is used for interpretation. This allows the direction, strength, and symmetry of (in)congruence effects to be examined simultaneously (Shanock et al., 2010; Nestler et al., 2019). Specifically, we based our procedure on the multilevel approach described by Berg et al. (2023) and adapted it to a Bayesian framework informed by Hu and Ram (2025).

A third model was performed to test hypothesis 3, using Bayesian multivariate multilevel polynomial regression, once the necessary preparations had been made (a detailed description can be found in the Appendix). In line with Berg et al. (2023), we included the untransformed, group-mean-centered predictors team goal commitment and team goal commitment of others at the start of the season, plus team-level means were used as between-level controls. Additionally, the three outcomes (RC, TC, and PC 2 months later) were included on their original scales, which allowed for residual correlations between the outcomes. To account for right skewness in the three outcome variables, the model was estimated using a Student-t likelihood. Cases with missing values were excluded from the data for the PR-model, only using complete cases for estimation. Furthermore, the PR-Model priors are analogous to those of the other two models and are reported in Supplemental material B. Finally, the model estimated Bayesian inference via HMC sampling, running eight chains of 5,000 warmup iterations and 10,000 post-warmup iterations per chain, yielding 80,000 posterior samples in total. Thereafter, we conducted a series of diagnostic checks as described above.

After fitting the model to the data, we estimated the RS parameters and created three-dimensional plots using the regression parameters. These RS parameters are fitted into a coordinate cube to create one three-dimensional plot per conflict type. Thus, the *X* (personal team goal commitment), *Y* (perceived team goal commitment of other team members), and *Z* (perceived level of conflict) variables are represented by the cube’s axes (Berg et al., 2023).

After visually representing the estimated RS parameters in a three-dimensional plot, (in)congruence between team goal commitment (*X*) and team goal commitment of the others (*Y*) and their joint prediction of the perceived level of conflict type (*Z*) can be examined using the estimated response surface. On the one hand, congruence is represented by the line of congruence (LOC; see Berg et al., 2023; Nestler et al., 2019), which reflects the model-predicted level of conflict type (*Z*) for team members whose team goal commitment is perfectly congruent to their perceived team goal commitment of the others (i.e., *X* = *Y*). On the other hand, incongruence is represented by the line of incongruence (LOIC), which reflects the model-predicted level of conflict type (*Z*) for team members whose team goal commitment is equal in magnitude but opposite in sign compared to their perceived team goal commitment of the others (i.e., *X* = −*Y*).

Both the LOC and the LOIC are defined by their respective response surface parameters. First, the LOC parameter *â*_1_ represents the linear slope when *X* and *Y* match, while *â*_2_ indicates whether the LOC is linear (if *â*_2_ = 0) or curvilinear (if *â*_2_ ≠ 0). That is, for example, if *â*_1_ > 0 means that the predicted conflict level is higher when personal team goal commitment and perceived team goal commitment of others are at a high level than when they agree at a lower level. Further, as another example, an *â*_2_ < 0 indicates that the model predicts the highest conflict level for a specific congruent combination for personal team goal commitment and perceived team goal commitment of others, while the conflict level is predicted to be lower for all other congruence combinations of team goal commitment and team goal commitment of others. Second, the LOIC parameter *â*_3_ represents the slope of the LOIC, while *â*_4_ indicates whether the LOIC is linear (if *â*_4_ = 0) curvilinear (if *â*_4_ ≠ 0). For example, when *â*_3_ = 0, this could mean that team members whose team goal commitment and perceived team goal commitment of others are equal in magnitude but opposite in sign, are predicted to have the lowest predicted conflict level. However, predicted conflict level is higher for team members whose personal team goal commitment exceeds the perceived team goal commitment of the others (i.e., *X* > *Y*) and for team members whose personal team goal commitment falls below the perceived team goal commitment of the others (i.e., *X* < *Y*). Furthermore, exemplary, an *â*_4_ > 0 indicates a U-shape for the LOIC, with having a lowest point of conflict level and increasing symmetrically at both sides of this point, this means that the higher the discrepancy between the predictors, the higher the conflict level. Additional to the LOC/LOIC-parameters, the response surface parameter *â*_5_ for the first principal axis (FPA) indicates the general curvature of the surface independent of LOC and LOIC (Berg et al., 2023; Nestler et al., 2019).

Following Berg et al. (2023), two approaches currently exist for interpreting RS parameters and plots. On the one hand, the approach described by Shanock et al. (2010) depicts that RS parameters could be interpreted in isolation, while on the other hand the approach described by Nestler et al. (2019) argue that all of the described RS parameters should be considered together when interpreting the outcome-prediction by an (in)congruence. That is, that (a) the LOC is flat (*â*_1_ = 0, *â*_2_ = 0), (b) the LOIC shows an inverted U-shape centered at the origin (*â*_3_ = 0, *â*_4_ < 0), and (c) the first principal axis equals the LOC.

## 3 Results

### 3.1 Descriptive statistics

Table 1 shows the descriptive statistics for the study variables. In general, the average level of the predictors goal compatibility, team goal commitment and team goal commitment of the other team members was above the midpoint of midpoint of their respective scales. Regarding the conflict scales, the average level was below the midpoint of the respective scale at the start of the season (baseline) and 2 months later (outcome). The dispersion of all variables was high within the total sample. However, the minimum scores on the team goal commitment scales were not reported. The maximum score on the conflict scale was also rarely reported.

**Table 1:** Descriptive statistics for team conflict and team goal commitment variables.

	*M*	*SD*	Minimum	Maximum
**Predictor Variables (T1)**				
Goal Compatibility ^a^	11.91	2.75	1	14
Team Goal Commitment ^b^	4.43	0.71	1.80	5.00
Team Goal Commitment of Others ^b^	4.12	0.70	1.80	5.00
**Baseline Variables (T1)**				
Relationship Conflict ^c^	2.84	1.22	1.00	7.50
Task Conflict ^c^	3.54	1.43	1.00	8.33
Process Conflict ^c^	3.50	1.45	1.00	9.00
**Outcome Variables (T2)**				
Relationship Conflict ^c^	2.92	1.31	1.00	7.00
Task Conflict ^c^	3.65	1.45	1.00	7.67
Process Conflict ^c^	3.44	1.42	1.00	7.50

*n*_T1_ = 356*. n*_T2_ = 173. ^a^ range: 1-14. ^b^ range: 1-5. ^c^ range: 1-9.

### 3.2 Prediction of conflict level

Table 2 displays the full results of Model 1 and Model 2. In Bayesian estimation, 95% credible intervals (CrI, reported in square brackets) that do not encompass zero are assumed to be significant. Thus, they can be used analogously to frequentist confidence intervals, providing inference reasoning (Hox et al., 2018), despite their differing philosophical bases (VanderPlas, 2014). The results of Model 1 showed that perceived team goal commitment of others at the start of the season was a significant negative predictor of perceived process conflict 2 months later (*β*_PC_ = 0.60, 95% CrI [0.10, 1.09]; reflect-log transformed). Furthermore, being a female team member in comparison to male team members was a significant negative predictor for relationship conflict (*β*_Gender_ = −0.44, 95% CrI [−0.83, −0.06]) and process conflict (*β*_Gender_ = −0.55, 95% CrI [−0.93, −0.16]). Apart from that, the results show that goal compatibility and personal team goal commitment did not correlate with conflict. After adding the baseline conflict levels, the results of Model 2 showed that the perceived level of team conflict was a significant positive predictor of perceived team conflict 2 months later (*β*_RC_ = 0.23, 95% CrI [0.08, 0.38]; *β*_TC_ = 0.29, 95% CrI [0.16, 0.43]; *β*_PC_ = 0.41, 95% CrI [0.29, 0.53]). Moreover, the same gender-specific predictions remained significant as in Model 1 for Model 2; relationship conflict (*β*_Gender_ = −0.50, 95% CrI [−0.89, −0.12]) and process conflict (*β*_Gender_ = −0.61, 95% CrI [−0.98, −0.23]). However, the negative correlation between team goal commitment of others and process conflict disappears; the other predictors remain insignificant, too. The lower LOOIC = 1523.7 (SE = 41.7) indicated a better fit for Model 2 than the LOOIC = 1564.8 (SE = 40.2) for Model 1.

**Table 2:** Bayesian multivariate multilevel models predicting team conflict (ICS-S) at T2.

**Outcome: Conflict Type (T2)**	Model 1				Model 2		
	Relationship	Task	Process		Relationship	Task	Process
**Fixed effects**							
Predictor (T1)	Coefficient [95% CrI]	Coefficient [95% CrI]	Coefficient [95% CrI]		Coefficient [95% CrI]	Coefficient [95% CrI]	Coefficient [95% CrI]
Intercept	2.99 [2.72, 3.27]	3.67 [3.36, 3.97]	3.52 [3.24, 3.80]		3.02 [2.75, 3.31]	3.70 [3.40, 3.99]	3.57 [3.30, 3.82]
Baseline ^a^	—	—	—		**0.23** **[0.08, 0.38]**	**0.29** **[0.16, 0.43]**	**0.41** **[0.29, 0.53]**
Age ^a^	0.00 [-0.03, 0.04]	0.01 [-0.03, 0.05]	0.01 [-0.03, 0.05]		0.00 [-0.03, 0.04]	0.01 [-0.03, 0.05]	0.01 [-0.02, 0.05]
Gender ^b^	**-0.44** **[-0.83, -0.06]**	-0.39 [-0.81, 0.05]	**-0.55** **[-0.93, -0.16]**		**-0.50** **[-0.89, -0.12]**	-0.42 [-0.84, 0.02]	**-0.61** **[-0.98, -0.23]**
Goal Compatibility ^a, c^	0.11 [-0.33, 0.54]	-0.12 [-0.62, 0.37]	-0.19 [-0.67, 0.28]		0.03 [-0.40, 0.46]	-0.10 [-0.58, 0.38]	-0.13 [-0.56, 0.30]
Team Goal Commitment ^a, c^	-0.26 [-0.89, 0.37]	-0.10 [-0.78, 0.58]	-0.29 [-0.94, 0.37]		-0.27 [-0.89, 0.35]	-0.16 [-0.84, 0.52]	-0.29 [-0.93, 0.35]
Team Goal Commitment of Others ^a, d^	0.24 [-0.22, 0.70]	0.26 [-0.26, 0.78]	**0.60** **[0.10, 1.09]**		0.19 [-0.27, 0.65]	0.23 [-0.27, 0.74]	0.36 [-0.10, 0.82]
**Random effects**							
σ_Person:Intercept_	0.20 [0.01, 0.53]	0.32 [0.02, 0.75]	0.34 [0.02, 0.70]		0.19 [0.01, 0.51]	0.36 [0.02, 0.79]	0.26 [0.01, 0.61]
σ_Team:Intercept_	0.32 [0.06, 0.59]	0.35 [0.06, 0.63]	0.27 [0.03, 0.53]		0.33 [0.07, 0.59]	0.34 [0.05, 0.63]	0.31 [0.07, 0.55]
σ_Team:Goal Compatibility_^a, c^	0.08 [0.00, 0.23]	0.08 [0.00, 0.23]	0.08 [0.00, 0.23]		0.08 [0.00, 0.22]	0.08 [0.00, 0.23]	0.08 [0.00, 0.23]
σ_Team:Team Goal Commitment_^a, c^	0.39 [0.02, 1.03]	0.43 [0.02, 1.15]	0.37 [0.02, 1.00]		0.38 [0.02, 1.01]	0.50 [0.02, 1.26]	0.59 [0.03, 1.29]
σ_Team:Team Goal Commitment of Others_^a, d^	0.21 [0.01, 0.60]	0.31 [0.01, 0.83]	0.28 [0.01, 0.76]		0.21 [0.01, 0.60]	0.29 [0.01, 0.79]	0.24 [0.01, 0.65]
**Model fit**							
Bayesian R² [95% CrI]	0.15 [0.05, 0.31]	0.19 [0.07, 0.39]	0.21 [0.09, 0.39]		0.19 [0.08, 0.35]	0.28 [0.13, 0.50]	0.35 [0.22, 0.51]
LOOIC (SE)		1564.8 (40.2)				1523.7 (41.7)	

CrI = credible intervals. Baseline = respective conflict type at T1. ^a^ group-mean centered. ^b^ 0 = male, 1 = female. ^c^ reflect and inverse transformed. ^d^ reflect and logarithm transformed. Bold values indicate fixed effects which do not encompass zero.

### 3.3 Association between (in)congruence of team goal commitment and conflict level

Table 3 displays the full results of Model 3. In line with Model 1, the results showed that perceived team goal commitment of others at the start of the season was a significant negative predictor of perceived process conflict 2 months later (*β*_PC_ = −0.41, 95% CrI [−0.76, −0.06]). Apart from that, the results showed that goal compatibility, personal team goal commitment and their respective squared terms did not correlate with conflict level after 2 months. Furthermore, we found the surface parameter *â*_1_ to be significant for the explanation of process conflict (*â*_1_ = −0.48, 95% CrI [−0.83, −0.14]). Following Shanock et al.'s (2010) approach to interpreting significant parameters, the *â*_1_ parameter showed a negative association between congruence of team goal commitment and team goal commitment of others on process conflict. Specifically, when both team goal commitment and team goal commitment of others are high, process conflict is low, and vice versa. However, according to Nestler et al. (2019), parameters cannot be interpreted in isolation. Thus, our results would not allow us to draw any conclusions about prediction by (in)congruence using this approach. Additionally, Figure 1 shows the three-dimensional plot of process conflict, which fits the RS parameters into a coordinate cube. In line with the significant surface parameter, the figure shows a downward trend along the LOC (and a flat shape along the LOIC). Three-dimensional plots of the other conflict types are not reported, as no significant surface parameters were found for them (see also Table 3).

**Table 3:** Bayesian multivariate multilevel polynomial regression coefficients and “average” surface parameters.

		Model 3		
**Outcome: Conflict Type (T2)**		Relationship	Task	Process
**Fixed effects**				
Predictor (T1)		Coefficient [95% CrI]	Coefficient [95% CrI]	Coefficient [95% CrI]
Intercept		3.39 [0.13, 6.66]	3.77 [0.34, 7.21]	3.44 [0.16, 6.66]
Team Goal Commitment ^a^ (X)		-0.06 [-0.42, 0.30]	-0.04 [-0.45, 0.38]	-0.07 [-0.46, 0.32]
Team Goal Commitment of Others ^a^ (Y)		-0.17 [-0.47, 0.14]	-0.19 [-0.56, 0.18]	**-0.41 [-0.76, -0.06]**
X²		0.03 [-0.40, 0.49]	-0.13 [-0.59, 0.33]	0.08 [-0.36, 0.52]
X × Y		0.14 [-0.37, 0.65]	0.05 [-0.53, 0.62]	-0.01 [-0.56, 0.55]
Y²		-0.21 [-0.55, 0.11]	-0.03 [-0.44, 0.39]	-0.00 [-0.41, 0.41]
Mean Team Goal Commitment		0.03 [-0.94, 0.99]	0.06 [-0.90, 1.04]	-0.06 [-1.02, 0.92]
Mean Team Goal Commitment of Others		0.03 [-0.95, 0.99]	0.07 [-0.91, 1.04]	-0.10 [-1.08, 0.87]
Mean X²		0.00 [-0.40, 0.41]	-0.03 [-0.44, 0.39]	0.17 [-0.23, 0.58]
Mean X × Y		0.00 [-0.79, 0.79]	0.02 [-0.78, 0.82]	-0.03 [-0.83, 0.76]
Mean Y²		-0.05 [-0.51, 0.41]	-0.04 [-0.51, 0.42]	-0.13 [-0.59, 0.32]
**Random effects**				
σ_Person:Intercept_		0.17 [0.01, 0.46]	0.33 [0.02, 0.75]	0.31 [0.02, 0.66]
σ_Team:Intercept_		0.32 [0.04, 0.60]	0.35 [0.07, 0.63]	0.17 [0.01, 0.44]
σ_Team:Team Goal Commitment c_ (X)		0.23 [0.01, 0.60]	0.29 [0.01, 0.76]	0.18 [0.01, 0.51]
σ_Team:Team Goal Commitment of Others c_ (Y)		0.15 [0.01, 0.42]	0.26 [0.01, 0.67]	0.21 [0.01, 0.56]
σ_Team:X²_		0.37 [0.02, 0.83]	0.19 [0.01, 0.54]	0.23 [0.01, 0.61]
σ_Team:X × Y_		0.23 [0.01, 0.65]	0.28 [0.01, 0.76]	0.34 [0.01, 0.90]
σ_Team:Y²_		0.17 [0.01, 0.48]	0.30 [0.01, 0.75]	0.36 [0.02, 0.81]
**Model fit**				
Bayesian R² [95% CrI]		0.18 [0.08, 0.30]	0.22 [0.10, 0.40]	0.21 [0.11, 0.36]
LOOIC (SE)			1589.1 (43.5)	
**“Average” surface parameters**				
LOC	*â*_1_	-0.23 [-0.54, 0.09]	-0.23 [-0.60, 0.15]	**-0.48 [-0.83, -0.14]**
	*â*_2_	-0.04 [-0.44, 0.36]	-0.11 [-0.58, 0.33]	0.07 [-0.36, 0.51]
LOIC	*â*_3_	0.11 [-0.36, 0.57]	0.15 [-0.39, 0.70]	0.35 [-0.17, 0.86]
	*â*_4_	-0.32 [-1.10, 0.47]	-0.21 [-1.07, 0.67]	0.08 [-0.76, 0.93]
FPA	*â*_5_	0.24 [-0.22, 0.72]	-0.10 [-0.64, 0.43]	0.08 [-0.44, 0.59]

CrI = credible intervals; LOC = line of congruence; LOIC = line of incongruence; FPA = first principal axis. ^a^ group-mean centered. Bold values indicate fixed effects and surface parameters which do not encompass zero.

**Figure 1:**
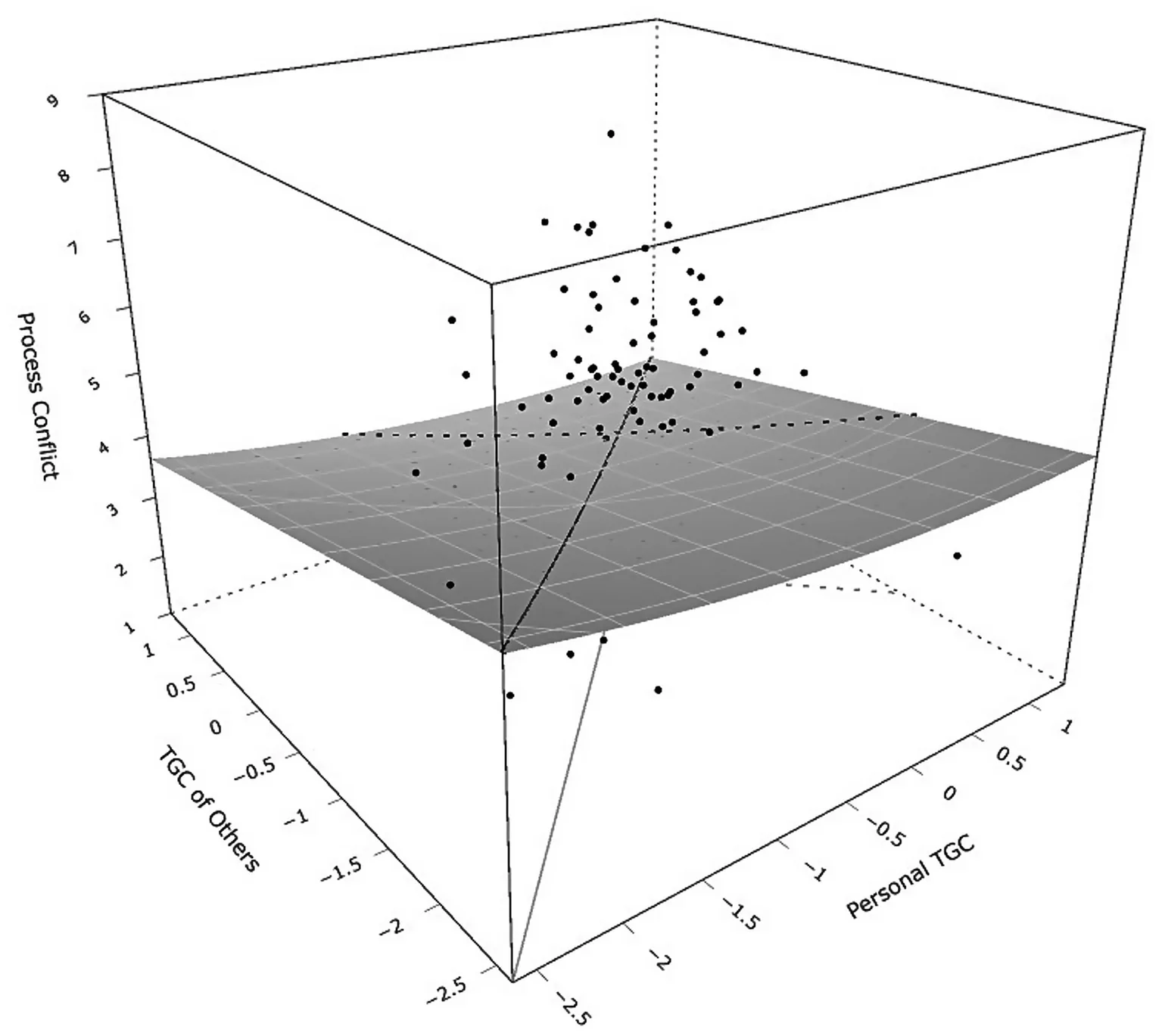
Response surface plot for process conflict (T2) as predicted by personal team goal commitment (T1) and team goal commitment of the other team members (T1). TGC = Team goal commitment. Line of congruence (LOC) is represented by the continuous line on the surface; line of incongruence (LOIC) is represented by the dotted line on the surface; both lines are also mirrored at the bottom of the cube. Each black dot within the cube represents an individual team member’s response; the surface hides the dots beneath it.

## 4 Discussion

The primary aim of this study was to longitudinally examine whether team goal commitment influences team conflict over time. In the present study, members of women’s and men’s amateur soccer teams completed questionnaires at the beginning of the season (T1) and 2 months later (T2). The study showed that (A) the higher the initial perceived level of conflict at the start of the season, the higher the perceived level of conflict 2 month later. (B) With regard to the study objective, in the first step of the analysis, it seems, preliminary, as if a negative correlation was found between the extent to which players assessed their team members’ team goal commitment at the start of the season and their own perception of team conflict (process conflict) after 2 months. However, this correlation no longer appears once the initial level of process conflict at the start of the season is taken into account in the analysis. Furthermore, neither the alignment of individual goals with team goals nor individual team goal commitment at the start of the season predicted team conflict 2 months later. (C) Furthermore, it was found that the level of relationship conflict and process conflict was perceived more strongly by members of men’s teams than by members of women’s teams. (D) Finally, the exploratory polynomial regression with response surface method indicated a negative effect of a congruence between individual team goal commitment and the perceived team goal commitment of the other team members on process conflict.

(A) The perceived level of team conflict among amateur soccer players at the start of the season positively correlates with their perception of team conflict 2 months later. The higher the perceived initial level at the start of the season, the higher the conflict level after 2 months across the three types of conflict examined (relationship, task, and process conflict). From the start of the season, players have different individual orientations towards team conflict, including how they “frame conflict situations, their pre-existing beliefs about conflict and their social motives within conflict situations” (Krueger et al., 2022), which have a relatively stable influence on the perceived level of conflict at the start of the season and 2 months later. Furthermore, it can be assumed that intra- and interpersonal factors, such as intrapersonal tension (Lewin, 1948) or the involvement of team members in possible interpersonal conflict constellations (Jehn et al., 2013), could explain the correlated perception of team conflict at the start of the season and 2 months later. Moreover, the correlation may indicate that conflicts have a stability component (Shah et al., 2021). Once conflicts exist, there is a high chance that they will remain, and conversely, it is very likely that the absence of conflicts will persist.

(B) When initial team conflict at the start of the season is not controlled for, lower perception of the team goal commitment of other team members at the beginning of the season appears to be associated with a higher level of process conflict later in the season. However, the negative correlation between team goal commitment of others and process conflict disappears, once the initial level of process conflict at the start of the season is taken into account in the analysis. This can be explained in terms of both statistics and content. In statistical terms, the association between team goal commitment of the other team members and process conflict is considerably weakened. The credible interval includes zero, indicating little evidence for an association beyond shared variance with simultaneously measured conflict levels. In terms of content, it is plausible that the two constructs are intertwined at the perceptual level. Team goal commitment of the other team members reflects how strongly team members perceive others as being determined to pursue the team goal (e.g., “My team members are strongly committed to pursuing our team goal”), whereas process conflict refers to perceptions of disagreement within the team about who should do what (e.g., “To what extent do you disagree about the way to do things in your team?”). Thus, the attenuation of the association between team goal commitment of the others and process conflict (T2) after controlling for initial levels of process conflict (T1) may reflect differences as well as linkages in the evaluation of perceptions: whereas process conflict represents an assessment of the team as a whole, perceptions of others’ goal commitment reflect a subcomponent of a broader team-level evaluation (i.e., assessment of the others). Moreover, while team goal commitment of the others remains more at a cognitive level, process conflict encompasses not only cognitive disagreements but also affective tensions (e.g., “To what extent is there tensions in your team caused by not completing their roles?”). This may intensify evaluative judgments of the team context and their effects. Consequently, when controlling for initial levels of process conflict at the beginning of the season, team members could perceive both the others as less committed to the team goal as well as the team in disagreement and tension about the fulfillment of tasks, and because of its intensity and broader nature the latter may absorb the former in its association with process conflict level 2 months later.

It is still interesting that, without controlling for baseline perceptions of team conflict, team members’ perceptions of the team goal commitment of others appear to be negatively associated with process conflict. The perceived low team goal commitment of other team members involves a negative perception of them, as they are perceived as uncommitted and as a possible limitation for the personal expectancy of success (Weldon and Weingart, 1993). As a result, team members who perceive low team goal commitment of other team members may experience negative emotional reactions and potential interference with the achievement of their own goals; both of which are ingredients for conflict (Barki and Hartwick, 2004). Moreover, this could create negative attitudes among team members (e.g., less trust and openness among each other), which may lead to a decline in group cohesion (Forsyth, 2021) and an increase in the likelihood of conflict (Korsgaard et al., 2014). Specifically for process conflict, low team goal commitment of others may mean that team members perceive that the other team members are not contributing to the processes and strategies for achieving the common goal; rather, they could even perceive that the other team members are hindering these efforts (Jehn and Mannix, 2001).

Furthermore, the study shows that the similarity between individual goals and the team goal (i.e., goal compatibility), as well as individual team goal commitment at the start of the season, have no influence on the perceived level of team conflict after 2 months. Since an agreement of individual and team goals contributes to identifying as a team member in team sports (Eys and Beauchamp, 2025), it was expected that goal compatibility would be negatively related to conflict, which we did not find. As a reason for this, we found that most players report (very) high levels of compatibility between their individual goals and the team goal. This creates a ceiling effect, meaning that the data is not suitable for investigating this effect. Not quite as extreme, but in a similar way, this could also be assumed for individual team goal commitment at the start of the season, which tends to be reported as high.

(C) In addition to the main effects, control variables (i.e., age and gender) were also taken into account in the analyses, thereby providing further possible explanations for the results. It was shown, that 2 months after the start of the season (T2) members of women’s teams report lower levels of relationship conflict and process conflicts than members of men’s teams. These differences between women’s and men’s teams do not apply to task conflicts. Krueger et al. (2022) and Weingart et al. (2015) describe that gender, among other factors, influences how conflict behavior is interpreted and that this is at least partly influenced by social expectations and norms regarding gender. For example, the authors describe that the expression of anger is more socially expected of men than women; these expectations often lead women to adjust their behavior, as not conforming results in a more negative evaluation than when a man expresses anger. The results of our study may suggest that gender-specific conflict behavior could also occur in the sports context (with predominantly single-gender groups), but that the topic or type of conflict might also plays a decisive role. In our study, members of men’s teams reported a higher perception of relationship and process conflicts than members of women’s teams. However, task conflicts were perceived similarly. This may show that gender-specific differences in conflicts do not exist per se and that task-related disagreements seem to be comparable across genders. In addition to the different interpretations of conflict behavior, future research on gender differences in team conflict (in sports) could also examine whether more constructive and cooperative conflict management approaches are chosen in women’s teams than in men’s teams, where more competitive and destructive approaches might be expected, based on the results of non-sports studies on gender differences in conflict behavior (Davis et al., 2010; Holt and DeVore, 2005; Rahim and Katz, 2019).

(D) We explored that, when the initial levels of team conflict at the start of the season were not taken into account, congruence between the perception of personal team goal commitment and that of the other team members could play a role for process conflict. Although these preliminary results are of limited robustness, there are two congruence constellations in our exploratory analysis that seem interesting to consider. Congruence between high levels of personal team goal commitment and high perceptions of the team goal commitment of others seems to be associated with low process conflict potential. When players perceive team goal commitment as high for themselves and for others, these players may perceive that they and the other team members are striving towards the team goal. This shared striving may be explained, among others, by high group cohesion and strong identification with the team, which are both associated with low conflict potential. More specifically, the former means that players might have a congruent perception of high team goal commitment when they perceive themselves as united with their team members in the pursuit of common goals (Carron, 1982). Furthermore, the latter means that team members could perceive a congruence of high team goal commitment when they identify strongly with the team, as this contributes positively to their self-concept (Tajfel and Turner, 1979).

However, congruence also means that both are perceived as low. That is, congruence between low personal team goal commitment and low perception of the team goal commitment of the others seems to be associated with a higher potential for process conflict. When players perceive team goal commitment as low for themselves and for others, they perceive themselves as less committed than other team members. At the same time, they perceive the team goal commitment of the other team members as lower relative to the mean perception of the team. In other words, these players do not aspire to the team goal, and simultaneously perceive that the rest of the team does not either, which differs from how their team members on average perceive each other. Players who perceive team goal commitment as low for themselves and for others, relative to the mean perception in their team, probably do not feel integrated into the team and possibly have a divergent negative perception of the team, which both are associated with an increased potential for conflict.

Against our expectations, an incongruence in the perception of personal team goal commitment and that of others does not play a role for conflict. We had expected higher perceived team goal commitment discrepancy (i.e., personal team goal commitment perceived as higher than others’ team goal commitment and/or others’ team goal commitment perceived as higher than personal team goal commitment) to be associated with higher conflict potential, but this was not the case in our exploratory analysis. One reason for this could be statistical in nature, namely the LOIC being too narrow due to a lack of variance.

### 4.1 Strengths and limitations

The strengths of this study lie in its design, the setting, the assessment of goal commitment, and the originality of the research question. The first strength of this study lies in its longitudinal design, which allows for the examination of conflict that goes beyond the findings of cross-sectional studies. The sports setting (i.e., soccer) also allows for the study of a population in which teams are homogenous in terms of gender, enabling clear gender-specific effects to be examined (coming with the disadvantage that heterogenous groups cannot be taken into account). Furthermore, this is the first study to consider not only the individual team goal commitment of the respective team members, but also how they assess the rest of the team in this regard; this makes it possible, among other things, to examine (outcome) dependency in sports teams more closely (Papineau and Eys, 2025). Another strength is the originality of the research question, as it has not yet been explored. In addition to these strengths, this study has five general limitations relating to the sample, design, measurements, data source and the setting. First, the homogeneous groups are not only a strength but also represent a limitation, as the results are difficult to transfer to contexts with mixed-gender groups (e.g., the work context). Another limitation regarding the sample is the high dropout rate. Although the same teams were examined at both time points, their rosters changed during the study period. Additionally, some players were simply absent at the second measurement point. This is particularly common in voluntary amateur sports, as athletes may lack motivation to train or have other responsibilities outside of sports. We also tested whether dropouts could be systematically explained by initial levels of team conflict or team goal commitment, but no relation was found (detailed results can be found in the Appendix). Moreover, a further limitation regarding the sample is that not all players had a clear understanding of their team’s goal. In this context, subsequent reanalyzes—which included only those players who reported that their team had a specific team goal—show that the results of Model 1 and Model 2 remain valid. However, in the reanalysis of Model 3, no significant predictors can be identified any longer. This means that a subsequent response surface analysis is no longer suitable for this model. Second, the study design does not allow causal interpretations since the main mechanisms were not controlled. For example, we are not aware of any situations during the study period that were characterized by conflict at that time. Third, the questionnaire was conducted as a self-report measure instead of observing objective behavior (especially conflict). Fourth, in our analysis, we only considered the players’ individual perceptions as a data source. Consequently, both the predictors and the outcomes were reported by the same player, which may have inflated their associations. Furthermore, specifically regarding perceived team goal commitment of the other team members, we did not consider the other team members’ actual commitment when predicting team conflict. Fifth, due to the setting, the findings only refer to amateur soccer teams and cannot be directly generalized to other sports or other team settings outside of sports.

### 4.2 Implications for research and practice

Implications for research derived from our study relate to the understanding of team goal commitment and team conflict. First, this study found initial evidence that, in addition to one’s own team goal commitment, the individual’s perception of the team goal commitment of other team members must also be taken into account in order to assess group performance and processes (Weldon and Weingart, 1993). However, the present study found no predictive evidence that the perception of other team members’ team goal commitment leads to team conflict when the initial level of team conflict is controlled. Without this control, a correlation with process conflict was found. This still does not provide causal evidence, but it shows that team conflict is not independent of the perception of other team members’ commitment to the team’s goals (and vice versa). In summary, the results of the study reflect the current state of research in that they suggest a relationship between team goal commitment and team conflict, but ultimately do not prove causation. Future studies should especially examine the interaction between perceived team goal commitment of others and process conflict in more detail. Second, the causes of conflict should be examined more specifically in relation to the type of conflict. This study reconfirms that different antecedents lead to different types of conflict, which shows how important it is to take this into account in future research projects with a theoretical basis (Greer and Dannals, 2017; Jehn, 2014). While the present study examined team conflict perception at the individual level, future studies should examine how team conflict develops across different levels (Korsgaard et al., 2008) and within the interpersonal dynamics of the team (e.g., social relations and social network approaches; Evans and Benson, 2025; Park et al., 2020). When planning future studies, care could also be taken to ensure that only completely newly formed teams are included and that they are recorded over a longer period of time with more measurement points, as this would allow both conflict development and complete group development to be recorded and examined. A comprehensive examination of group development would make it possible to examine both the (shared) experiences and conflict actions of team members and their impact on team conflict (Cronin and Bezrukova, 2019), as well as potentially conflictual situations in early stages of group development (Smith, 2001), which in turn would provide insights into conflict development. Alternatively, future studies could also look specifically at potentially conflictual situations (in soccer, for example, this would be teams fighting relegation or outside of sports, experimentally examine certain conflictual discussions). In addition, consideration should be given to examining other team sports as well as individual sports, gender-mixed teams and teams outside of sports, as the results offer transfer potential in other contexts. Furthermore, the suggested gender differences must be further investigated in future research.

Four implications can be drawn from the study for practical application. First, in team sports, the individual goals of players are in most cases highly compatible with the team goal, and personal commitment to the team goal is also usually high. Therefore, incompatibilities between individual and team goals, as well as signs of low team goal commitment, should be taken seriously. Coaches, sports psychologists, or leading players in positions of responsibility should contact the individual players and work together to find solutions or provide further help if it becomes apparent that the person concerned needs it. Second, it is important that team members have a realistic view of each other’s team goal commitment. This requires, for example, goal workshops, clear communication among each other, and clear messages from the coaches (Widmeyer and Ducharme, 1997; Senécal et al., 2008). Third, our data show that if conflicts arise at the start of the season, there is a high chance that they will remain. Thus, diagnostics and screenings should be carried out from the start of the season in order to enable a quick response. Fourth, if a high level of team conflict is perceived within the team, clear conflict management strategies have to be developed. If the perceived team conflict is not managed early on, it is likely to still have high levels of conflict 2 months later (Greer et al., 2008).

## 5 Conclusion

This study examined the relationship between team goal commitment and the perception of team conflict in amateur soccer teams over time. It was shown that the level of team conflict at the beginning of the season is an indicator of the level of team conflict 2 months later. Further research is needed to identify additional sources of team conflict. Future interventions for team conflict (in sports) should consider the individual baseline perception of team conflict, as well as team gender.

## Data Availability

The raw data supporting the conclusions of this article will be made available by the authors, without undue reservation.
