# Supplementary material for: Perception of team conflict and goal commitment over time: a multilevel analysis in sports teams
Source: Front Psychol. 2026 Oct 28;17:1935278. doi: 10.3389/fpsyg.2026.1935278 (PMC13752973; doi:10.3389/fpsyg.2026.1935278)
Supplement: Supplementary file 1 [file Supplementary_file_1.docx]

Appendix

## Preparation Steps for Multilevel Polynomial Regression and Response Surface Methodology

Following the guide by Berg et al. (2023), we executed necessary preparation steps prior to conducting multilevel polynomial regression (PR) and response surface methodology (RSM). First, we group-mean-centered our predictors team goal commitment and team goal commitment of others before constructing our models to obtain pure within-group estimates. This procedure is recommended when the data is collected at the same level of predictors and from the same sources (Preacher et al., 2006), which is the case with our data. In detail, all items were measured at the individual level, with each team member rating their personal team goal commitment and their perception of the team goal commitment of the other team members to the respective items. Second, following the recommendation of Shanock et al. (2010), we checked whether there were meaningful discrepancies between the predictors that would justify continuing with PR-RSM. We found that about 41.4% of the team members had significant different ratings for their personal team goal commitment and their perception of the team goal commitment of the other team members. This supported our approach to investigate whether and how the (in)congruence between team goal commitment and team goal commitment of the other team members at the start of the season is associated with the conflict types two months later. Third, we calculated item-level intraclass correlation (ICC) as an indicator to use multilevel models and checked whether adding the interaction between the two predictors and their squared terms would significantly improve the model, which only had the centered predictors. The ICC calculations resulted in ICC-values ranging from .16 to .26. These values indicate that 16% of the variance in process conflict, 20% of the variance in task conflict, and 26% of the variance in relationship conflict are attributable to team membership (Hox et al., 2018). Furthermore, the model comparison showed that adding the interaction between the two predictors and their squared terms increased the Bayesian R² by approximately 0.04 for both relationship and process conflict (95% credible intervals did not include zero), while slightly reducing the Bayesian R² for task conflict by approximately 0.01 (95% credible intervals entirely below zero). Overall, these results indicate that the explained variance (Bayesian R²) increases by about 4 percentage points for relationship and process conflict each, and decreases by about 1 percentage point for task conflict. Finally, we used leave-one-out cross-validation to check whether the polynomial approach improved predictive performance. The model comparison revealed that the polynomial model did not outperform the baseline model (expected log posterior density difference = −8.4, *SE* = 5.0). However, since the main objective of this analysis was a theory-driven examination of potential (in)congruence associations, we kept the polynomial model for interpretive purposes and relied on the previous models (Model 1 and Model 2) for predictive inference.

## Dropout Analysis

Table A1 shows the descriptive statistics and unpaired Welch t-test statistics for initial levels of team conflict and team goal commitment for each subgroup (completers and dropouts). The subgroups seem to be comparable in terms of the baseline variables at the start of the season. To test whether dropouts could be predicted by initial levels of team conflict or team goal commitment, a Bayesian multilevel binomial logistic regression model predicting dropout was estimated. The 95% credible intervals for the estimated associations between the baseline variables and study dropout included zero, suggesting little evidence of a meaningful association (see Table A2).

**TABLE A1** Baseline descriptive statistics for the completer and dropout subsamples

| Subsamples | Completer (*n* = 173) | | | | Dropout (*n* = 183) | | | |  |  |  |
| --- | --- | --- | --- | --- | --- | --- | --- | --- | --- | --- | --- |
|  | *M* | *SD* | Minimum | Maximum | *M* | *SD* | Minimum | Maximum | *t* ^c^ | *p* | Hedges' *g* ^d^ |
| Baseline Team Conflict (T1) |  |  |  |  |  |  |  |  |  |  |  |
| Relationship Conflict ^a^ | 2.73 | 1.14 | 1.00 | 7.50 | 2.94 | 1.30 | 1.00 | 7.00 | *t*(349.40) = -1.60 | .110 | -0.17 [-0.38; 0.04] |
| Task Conflict ^a^ | 3.53 | 1.37 | 1.00 | 7.00 | 3.56 | 1.49 | 1.00 | 8.33 | *t*(351.48) = -0.18 | .858 | -0.02 [-0.23; 0.19] |
| Process Conflict ^a^ | 3.50 | 1.41 | 1.00 | 8.00 | 3.50 | 1.49 | 1.00 | 9.00 | *t*(351.98) = -0.01 | .999 | -0.01 [-0.21; 0.21] |
| Baseline Team Goal Commitment (T1) |  |  |  |  |  |  |  |  |  |  |  |
| Team Goal Commitment ^b^ | 4.47 | 0.71 | 1.80 | 5.00 | 4.39 | 0.71 | 2.00 | 5.00 | *t*(352.93) = 1.09 | .276 | 0.12 [-0.09; 0.32] |
| Team Goal Commitment of Others ^b^ | 4.14 | 0.73 | 1.80 | 5.00 | 4.11 | 0.67 | 2.40 | 5.00 | *t*(347.55) = 0.41 | .684 | 0.04 [-0.16; 0.25] |

Completer = Players that were examined at the beginning of the season (Time 1) and two months later (Time 2). Dropout = Players that were only examined at the beginning of the season (Time 1). ^a^ range: 1-9. ^b^ range: 1-5. ^c^ t-test: unpaired Welch t-test. ^d^ 95% confidence interval.

**TABLE A2** Bayesian multilevel binomial logistic regression predicting dropout

| Outcome: Dropout ^a^ | Coefficient [95% CrI] |
| --- | --- |
|  | Fixed effects |
| Predictor (T1) |  |
| Intercept | 0.08 [-0.28, 0.44] |
| Relationship Conflict ^b^ | 0.19 [-0.11, 0.49] |
| Task Conflict ^b^ | -0.02 [-0.28, 0.23] |
| Process Conflict ^b^ | -0.07 [-0.33, 0.18] |
| Team Goal Commitment ^b, c^ | -0.37 [-1.14, 0.39] |
| Team Goal Commitment of Others ^b, d^ | -0.09 [-0.72, 0.54] |
|  | Random effects |
| σ _Person:Intercept_ | 0.52 [0.02, 1.36] |
| σ _Team:Intercept_ | 0.68 [0.35, 1.07] |
| σ _Team:Relationship Conflict_ ^b^ | 0.17 [0.01, 0.49] |
| σ _Team:Task Conflict_ ^b^ | 0.18 [0.01, 0.50] |
| σ _Team:Process Conflict_ ^b^ | 0.12 [0.01, 0.36] |
| σ _Team:Team Goal Commitment_ ^b, c^ | 0.41 [0.02, 1.14] |
| σ _Team:Team Goal Commitment of Others_ ^b, d^ | 0.32 [0.01, 0.90] |
|  | Model fit |
| Bayesian R² [95% CrI] | 0.17 [0.08, 0.33] |
| LOOIC (SE) | 486.9 (8.4) |

CrI = credible intervals. ^a^ 0 = Completer (players that were examined at the beginning of the season and two months later), 1 = Dropout (players that were only examined at the beginning of the season). ^b^ group-mean centered. ^c^ reflect and inverse transformed. ^d^ reflect and logarithm transformed. Bold values indicate fixed effects which do not encompass zero.

References

Berg, A.-K., Bakaç, C., and Kauffeld, S. (2023). Modeling (in)congruence in groups: A how-to guide for applying polynomial regression and response surface method to multilevel data. *Group Dyn. Theory Res. Pract.* 27, 154–170. doi: 10.1037/gdn0000200.

Hox, J. J., Moerbeek, M., and van Schoot, R. de (2018). *Multilevel analysis: Techniques and applications.* Third edition. New York, London: Routledge Taylor & Francis Group.

Preacher, K. J., Curran, P. J., and Bauer, D. J. (2006). Computational tools for probing interactions in multiple linear regression, multilevel modeling, and latent curve analysis. *J. Educ. Behav. Stat.* 31, 437–448. doi: 10.3102/10769986031004437.

Shanock, L. R., Baran, B. E., Gentry, W. A., Pattison, S. C., and Heggestad, E. D. (2010). Polynomial regression with response surface analysis: A powerful approach for examining moderation and overcoming limitations of difference scores. *J. Bus. Psychol.* 25, 543–554. doi: 10.1007/s10869-010-9183-4.
