# Supplementary material for: Perception of team conflict and goal commitment over time: a multilevel analysis in sports teams
Source: Front Psychol. 2026 Oct 28;17:1935278. doi: 10.3389/fpsyg.2026.1935278 (PMC13752973; doi:10.3389/fpsyg.2026.1935278)
Supplement: Supplementary file 2 [file Table_1.DOCX]

Supplementary Material

# 1 SUPPLEMENTAL MATERIAL A

**TABLE S1** Priors for Bayesian multivariate multilevel regression Model 1 and Model 2

| Parameters | Prior distribution | Prior Strength | Rationale | Hyperparameters |
| --- | --- | --- | --- | --- |
|  |  | Model 1 |  |  |
| RC Intercept | Normal(3.2,0.6) | Informative | The pilot data indicated that the mean relationship conflict values were slightly above the lower part of the 1–9 Likert scale. | μ=3.2, σ=0.6 |
| RC on Age ^a^ | Normal(0,0.1) | Weakly informative | The pilot indicated negligible associations between age and conflict outcomes. Therefore, a narrow prior centered at zero was used to reflect the small expected effects, while still allowing for updating. | μ=0, σ=0.1 |
| RC on Gender ^b^ | Normal(-0.6, 0.4) | Moderately informative | The pilot data suggested lower levels of relationship conflict among women than among men. Prior variance allows uncertainty while incorporating the observed pilot trend. | μ=-0.6, σ=0.4 |
| RC on Goal Compatibility ^a, c^ | Normal(0,0.5) | Weakly informative | Pilot data indicated minimal slope variation; prior constraints limit the parameter to a small but non-zero range, allowing the data to meaningfully update the posterior distribution. | μ=0, σ=0.5 |
| RC on Team Goal Commitment ^a, c^ | Normal(0,0.5) | Weakly informative | Pilot data indicated minimal slope variation; prior constraints limit the parameter to a small but non-zero range, allowing the data to meaningfully update the posterior distribution. | μ=0, σ=0.5 |
| RC on Team Goal Commitment of Others ^a, d^ | Normal(0,0.5) | Weakly informative | Pilot data indicated minimal slope variation; prior constraints limit the parameter to a small but non-zero range, allowing the data to meaningfully update the posterior distribution. | μ=0, σ=0.5 |
| RC on σ _Person:Intercept_ | Student-t(3,0,1) | Weakly informative | Based on the pilot data, the Student-t prior allows for heavier tails and accommodates heterogeneity among individuals. | ν=3, σ=1 |
| RC on σ _Team:Intercept_ | Student-t(3,0,0.6) | Moderately informative | Pilot ICC estimates revealed that there was less variability between teams than between individuals. Therefore, the prior reflects moderate team variance while avoiding extreme estimates. | ν=3, σ=0.6 |
| RC on σ _Team:Goal Compatibility_ ^a, c^ | Normal(0, 0.1) | Moderately informative | Pilot data indicated minimal slope variation; prior constraints limit the parameter to a small but non-zero range, allowing the data to meaningfully update the posterior distribution. | μ=0, σ=0.1 |
| RC on σ _Team:Team Goal Commitment_ ^a, c^ | Normal(0,0.5) | Weakly informative | Pilot data indicated minimal slope variation; prior constraints limit the parameter to a small but non-zero range, allowing the data to meaningfully update the posterior distribution. | μ=0, σ=0.5 |
| RC on σ _Team:Team Goal Commitment of Others_ ^a, d^ | Normal(0,0.5) | Weakly informative | Pilot data indicated minimal slope variation; prior constraints limit the parameter to a small but non-zero range, allowing the data to meaningfully update the posterior distribution. | μ=0, σ=0.5 |
| RC on σ _Residual_ | Student-t(5,0,0.9) | Weakly informative | Informed by pilot residual variance; permits realistic levels of within-person fluctuations. Student‑t prior allows heavier tails to accommodate possible outliers. | ν=5, σ=0.9 |
| TC Intercept | Normal(3.3,0.6) | Informative | The pilot data indicated that the mean task conflict values were slightly above the lower part of the 1–9 Likert scale. | μ=3.3, σ=0.6 |
| TC on Age ^a^ | Normal(0,0.1) | Weakly informative | The pilot indicated negligible associations between age and conflict outcomes. Therefore, a narrow prior centered at zero was used to reflect the small expected effects, while still allowing for updating. | μ=0, σ=0.1 |
| TC on Gender ^b^ | Normal(0,0.4) | Weakly informative | The pilot data suggested small but uncertain gender differences in conflict perceptions, so a symmetric prior with moderate spread was used to allow for potential effects without strong directional assumptions. | μ=0, σ=0.4 |
| TC on Goal Compatibility ^a, c^ | Normal(0,0.5) | Weakly informative | Pilot data indicated minimal slope variation; prior constraints limit the parameter to a small but non-zero range, allowing the data to meaningfully update the posterior distribution. | μ=0, σ=0.5 |
| TC on Team Goal Commitment ^a, c^ | Normal(0,0.5) | Weakly informative | Pilot data indicated minimal slope variation; prior constraints limit the parameter to a small but non-zero range, allowing the data to meaningfully update the posterior distribution. | μ=0, σ=0.5 |
| TC on Team Goal Commitment of Others ^a, d^ | Normal(0,0.5) | Weakly informative | Pilot data indicated minimal slope variation; prior constraints limit the parameter to a small but non-zero range, allowing the data to meaningfully update the posterior distribution. | μ=0, σ=0.5 |
| TC on σ _Person:Intercept_ | Student-t(3,0,1) | Weakly informative | Based on the pilot data, the Student-t prior allows for heavier tails and accommodates heterogeneity among individuals. | ν=3, σ=1 |
| TC on σ _Team:Intercept_ | Student-t(3,0,0.6) | Moderately informative | Pilot ICC estimates revealed that there was less variability between teams than between individuals. Therefore, the prior reflects moderate team variance while avoiding extreme estimates. | ν=3, σ=0.6 |
| TC on σ _Team:Goal Compatibility_ ^a, c^ | Normal(0, 0.1) | Moderately informative | Pilot data indicated minimal slope variation; prior constraints limit the parameter to a small but non-zero range, allowing the data to meaningfully update the posterior distribution. | μ=0, σ=0.1 |
| TC on σ _Team:Team Goal Commitment_ ^a, c^ | Normal(0,0.5) | Weakly informative | Pilot data indicated minimal slope variation; prior constraints limit the parameter to a small but non-zero range, allowing the data to meaningfully update the posterior distribution. | μ=0, σ=0.5 |
| TC on σ _Team:Team Goal Commitment of Others_ ^a, d^ | Normal(0,0.5) | Weakly informative | Pilot data indicated minimal slope variation; prior constraints limit the parameter to a small but non-zero range, allowing the data to meaningfully update the posterior distribution. | μ=0, σ=0.5 |
| TC on σ _Residual_ | Student-t(5,0,0.9) | Weakly informative | Informed by pilot residual variance; permits realistic levels of within-person fluctuations. Student‑t prior allows heavier tails to accommodate possible outliers. | ν=5, σ=0.9 |
| PC Intercept | Normal(3.6,0.6) | Informative | The pilot data indicated that process conflict levels were somewhat higher than those of other conflict types. | μ=3.6, σ=0.6 |
| PC on Age ^a^ | Normal(0,0.1) | Weakly informative | The pilot indicated negligible associations between age and conflict outcomes. Therefore, a narrow prior centered at zero was used to reflect the small expected effects, while still allowing for updating. | μ=0, σ=0.1 |
| PC on Gender ^b^ | Normal(0,0.4) | Weakly informative | The pilot data suggested small but uncertain gender differences in conflict perceptions, so a symmetric prior with moderate spread was used to allow for potential effects without strong directional assumptions. | μ=0, σ=0.4 |
| PC on Goal Compatibility ^a, c^ | Normal(0,0.5) | Weakly informative | Pilot data indicated minimal slope variation; prior constraints limit the parameter to a small but non-zero range, allowing the data to meaningfully update the posterior distribution. | μ=0, σ=0.5 |
| PC on Team Goal Commitment ^a, c^ | Normal(0,0.5) | Weakly informative | Pilot data indicated minimal slope variation; prior constraints limit the parameter to a small but non-zero range, allowing the data to meaningfully update the posterior distribution. | μ=0, σ=0.5 |
| PC on Team Goal Commitment of Others ^a, d^ | Normal(0,0.5) | Weakly informative | Pilot data indicated minimal slope variation; prior constraints limit the parameter to a small but non-zero range, allowing the data to meaningfully update the posterior distribution. | μ=0, σ=0.5 |
| PC on σ _Person:Intercept_ | Student-t(3,0,1) | Weakly informative | Based on the pilot data, the Student-t prior allows for heavier tails and accommodates heterogeneity among individuals. | ν=3, σ=1 |
| PC on σ _Team:Intercept_ | Student-t(3,0,0.6) | Moderately informative | Pilot ICC estimates revealed that there was less variability between teams than between individuals. Therefore, the prior reflects moderate team variance while avoiding extreme estimates. | ν=3, σ=0.6 |
| PC on σ _Team:Goal Compatibility_ ^a, c^ | Normal(0, 0.1) | Moderately informative | Pilot data indicated minimal slope variation; prior constraints limit the parameter to a small but non-zero range, allowing the data to meaningfully update the posterior distribution. | μ=0, σ=0.1 |
| PC on σ _Team:Team Goal Commitment_ ^a, c^ | Normal(0,0.5) | Weakly informative | Pilot data indicated minimal slope variation; prior constraints limit the parameter to a small but non-zero range, allowing the data to meaningfully update the posterior distribution. | μ=0, σ=0.5 |
| PC on σ _Team:Team Goal Commitment of Others_ ^a, d^ | Normal(0,0.5) | Weakly informative | Pilot data indicated minimal slope variation; prior constraints limit the parameter to a small but non-zero range, allowing the data to meaningfully update the posterior distribution. | μ=0, σ=0.5 |
| PC on σ _Residual_ | Student-t(5,0,0.9) | Weakly informative | Informed by pilot residual variance; permits realistic levels of within-person fluctuations. Student‑t prior allows heavier tails to accommodate possible outliers. | ν=5, σ=0.9 |
| Team RE correlations | LKJ(4) | Regularizing | The LKJ prior was used to reduce extreme correlations among random effects, while allowing for correlations if the data supported them. | η=4 |
| Residual correlations | LKJ(4) | Regularizing | Residual correlations between outcomes were allowed while shrinking extreme dependencies estimates. | η=4 |
|  |  | Model 2 (addition) |  |  |
| RC on Baseline ^a^ | Normal(0.5,0.3) | Moderately informative | Moderate stability of conflict measures between measurement occasions was indicated by Shah et al. (2021). | μ=0.5, σ=0.3 |
| TC on Baseline ^a^ | Normal(0.5,0.3) | Moderately informative | Moderate stability of conflict measures between measurement occasions was indicated by Shah et al. (2021). | μ=0.5, σ=0.3 |
| PC on Baseline ^a^ | Normal(0.5,0.3) | Moderately informative | Moderate stability of conflict measures between measurement occasions was indicated by Shah et al. (2021). | μ=0.5, σ=0.3 |
|  |  |  |  |  |

Default priors in brms were overridden by specified priors where indicated. **RC = relationship conflict**; **TC = task conflict**; **PC = process conflict**; RE = Random-Effects; LKJ = Lewandowski, Kurowicka, and Joe (initials of LKJ prior inventors). ^a^ **group-mean centered**. ^b^ 0 = male, 1 = female. ^c^ reflect and inverse transformed. ^d^ reflect and logarithm transformed.

# 2 SUPPLEMENTAL MATERIAL B

**TABLE S2** Priors for Bayesian multivariate multilevel polynomial regression Model 3

| Parameters | Prior distribution | Prior Strength | Rationale | Hyperparameters |
| --- | --- | --- | --- | --- |
| RC Intercept | Normal(3.2,0.6) | Informative | The pilot data indicated that the mean relationship conflict values were slightly above the lower part of the 1–9 Likert scale. | μ=3.2, σ=0.6 |
| RC on Team Goal Commitment ^a^ (X) | Normal(0,0.5) | Weakly informative | Pilot data indicated minimal slope variation; prior constraints limit the parameter to a small but non-zero range, allowing the data to meaningfully update the posterior distribution. | μ=0, σ=0.5 |
| RC on Team Goal Commitment of Others ^a^ (Y) | Normal(0,0.5) | Weakly informative | Pilot data indicated minimal slope variation; prior constraints limit the parameter to a small but non-zero range, allowing the data to meaningfully update the posterior distribution. | μ=0, σ=0.5 |
| RC on X² | Normal(0,0.5) | Weakly informative | For the polynomial regression terms, weakly informative priors were used to regularize higher-order effects while allowing the data to update the posterior. | μ=0, σ=0.5 |
| RC on X × Y | Normal(0,0.5) | Weakly informative | For the polynomial regression terms, weakly informative priors were used to regularize higher-order effects while allowing the data to update the posterior. | μ=0, σ=0.5 |
| RC on Y² | Normal(0,0.5) | Weakly informative | For the polynomial regression terms, weakly informative priors were used to regularize higher-order effects while allowing the data to update the posterior. | μ=0, σ=0.5 |
| RC on Mean Team Goal Commitment | Normal(0,0.5) | Weakly informative | For the polynomial regression terms, weakly informative priors were used to regularize higher-order effects while allowing the data to update the posterior. | μ=0, σ=0.5 |
| RC on Mean Team Goal Commitment of Others | Normal(0,0.5) | Weakly informative | For the polynomial regression terms, weakly informative priors were used to regularize higher-order effects while allowing the data to update the posterior. | μ=0, σ=0.5 |
| RC on Mean X² | Normal(0,0.5) | Weakly informative | For the polynomial regression terms, weakly informative priors were used to regularize higher-order effects while allowing the data to update the posterior. | μ=0, σ=0.5 |
| RC on Mean X × Y | Normal(0,0.5) | Weakly informative | For the polynomial regression terms, weakly informative priors were used to regularize higher-order effects while allowing the data to update the posterior. | μ=0, σ=0.5 |
| RC on Mean Y² | Normal(0,0.5) | Weakly informative | For the polynomial regression terms, weakly informative priors were used to regularize higher-order effects while allowing the data to update the posterior. | μ=0, σ=0.5 |
| RC on σ _Person:Intercept_ | Student-t(3,0,1) | Weakly informative | Based on the pilot data, the Student-t prior allows for heavier tails and accommodates heterogeneity among individuals. | ν=3, σ=1 |
| RC on σ _Team:Intercept_ | Student-t(3,0,0.6) | Moderately informative | Pilot ICC estimates revealed that there was less variability between teams than between individuals. Therefore, the prior reflects moderate team variance while avoiding extreme estimates. | ν=3, σ=0.6 |
| RC on σ _Team:Team Goal Commitment_ ^a^ (X) | Normal(0,0.5) | Weakly informative | Pilot data indicated minimal slope variation; prior constraints limit the parameter to a small but non-zero range, allowing the data to meaningfully update the posterior distribution. | μ=0, σ=0.5 |
| RC on σ _Team:Team Goal Commitment of Others_ ^a^ (Y) | Normal(0,0.5) | Weakly informative | Pilot data indicated minimal slope variation; prior constraints limit the parameter to a small but non-zero range, allowing the data to meaningfully update the posterior distribution. | μ=0, σ=0.5 |
| RC on σ _Team:X²_ | Normal(0,0.5) | Weakly informative | For the polynomial regression terms, weakly informative priors were used to regularize higher-order effects while allowing the data to update the posterior. | μ=0, σ=0.5 |
| RC on σ _Team:X × Y_ | Normal(0,0.5) | Weakly informative | For the polynomial regression terms, weakly informative priors were used to regularize higher-order effects while allowing the data to update the posterior. | μ=0, σ=0.5 |
| RC on σ _Team:Y²_ | Normal(0,0.5) | Weakly informative | For the polynomial regression terms, weakly informative priors were used to regularize higher-order effects while allowing the data to update the posterior. | μ=0, σ=0.5 |
| TC Intercept | Normal(3.3,0.6) | Informative | The pilot data indicated that the mean task conflict values were slightly above the lower part of the 1–9 Likert scale. | μ=3.3, σ=0.6 |
| TC on Team Goal Commitment ^a^ (X) | Normal(0,0.5) | Weakly informative | Pilot data indicated minimal slope variation; prior constraints limit the parameter to a small but non-zero range, allowing the data to meaningfully update the posterior distribution. | μ=0, σ=0.5 |
| TC on Team Goal Commitment of Others ^a^ (Y) | Normal(0,0.5) | Weakly informative | Pilot data indicated minimal slope variation; prior constraints limit the parameter to a small but non-zero range, allowing the data to meaningfully update the posterior distribution. | μ=0, σ=0.5 |
| TC on X² | Normal(0,0.5) | Weakly informative | For the polynomial regression terms, weakly informative priors were used to regularize higher-order effects while allowing the data to update the posterior. | μ=0, σ=0.5 |
| TC on X × Y | Normal(0,0.5) | Weakly informative | For the polynomial regression terms, weakly informative priors were used to regularize higher-order effects while allowing the data to update the posterior. | μ=0, σ=0.5 |
| TC on Y² | Normal(0,0.5) | Weakly informative | For the polynomial regression terms, weakly informative priors were used to regularize higher-order effects while allowing the data to update the posterior. | μ=0, σ=0.5 |
| TC on Mean Team Goal Commitment | Normal(0,0.5) | Weakly informative | For the polynomial regression terms, weakly informative priors were used to regularize higher-order effects while allowing the data to update the posterior. | μ=0, σ=0.5 |
| TC on Mean Team Goal Commitment of Others | Normal(0,0.5) | Weakly informative | For the polynomial regression terms, weakly informative priors were used to regularize higher-order effects while allowing the data to update the posterior. | μ=0, σ=0.5 |
| TC on Mean X² | Normal(0,0.5) | Weakly informative | For the polynomial regression terms, weakly informative priors were used to regularize higher-order effects while allowing the data to update the posterior. | μ=0, σ=0.5 |
| TC on Mean X × Y | Normal(0,0.5) | Weakly informative | For the polynomial regression terms, weakly informative priors were used to regularize higher-order effects while allowing the data to update the posterior. | μ=0, σ=0.5 |
| TC on Mean Y² | Normal(0,0.5) | Weakly informative | For the polynomial regression terms, weakly informative priors were used to regularize higher-order effects while allowing the data to update the posterior. | μ=0, σ=0.5 |
| TC on σ _Person:Intercept_ | Student-t(3,0,1) | Weakly informative | Based on the pilot data, the Student-t prior allows for heavier tails and accommodates heterogeneity among individuals. | ν=3, σ=1 |
| TC on σ _Team:Intercept_ | Student-t(3,0,0.6) | Moderately informative | Pilot ICC estimates revealed that there was less variability between teams than between individuals. Therefore, the prior reflects moderate team variance while avoiding extreme estimates. | ν=3, σ=0.6 |
| TC on σ _Team:Team Goal Commitment_ ^a^ (X) | Normal(0,0.5) | Weakly informative | Pilot data indicated minimal slope variation; prior constraints limit the parameter to a small but non-zero range, allowing the data to meaningfully update the posterior distribution. | μ=0, σ=0.5 |
| TC on σ _Team:Team Goal Commitment of Others_ ^a^ (Y) | Normal(0,0.5) | Weakly informative | Pilot data indicated minimal slope variation; prior constraints limit the parameter to a small but non-zero range, allowing the data to meaningfully update the posterior distribution. | μ=0, σ=0.5 |
| TC on σ _Team:X²_ | Normal(0,0.5) | Weakly informative | For the polynomial regression terms, weakly informative priors were used to regularize higher-order effects while allowing the data to update the posterior. | μ=0, σ=0.5 |
| TC on σ _Team:X × Y_ | Normal(0,0.5) | Weakly informative | For the polynomial regression terms, weakly informative priors were used to regularize higher-order effects while allowing the data to update the posterior. | μ=0, σ=0.5 |
| TC on σ _Team:Y²_ | Normal(0,0.5) | Weakly informative | For the polynomial regression terms, weakly informative priors were used to regularize higher-order effects while allowing the data to update the posterior. | μ=0, σ=0.5 |
| PC Intercept | Normal(3.6,0.6) | Informative | The pilot data indicated that process conflict levels were somewhat higher than those of other conflict types. | μ=3.6, σ=0.6 |
| PC on Team Goal Commitment ^a^ (X) | Normal(0,0.5) | Weakly informative | Pilot data indicated minimal slope variation; prior constraints limit the parameter to a small but non-zero range, allowing the data to meaningfully update the posterior distribution. | μ=0, σ=0.5 |
| PC on Team Goal Commitment of Others ^a^ (Y) | Normal(0,0.5) | Weakly informative | Pilot data indicated minimal slope variation; prior constraints limit the parameter to a small but non-zero range, allowing the data to meaningfully update the posterior distribution. | μ=0, σ=0.5 |
| PC on X² | Normal(0,0.5) | Weakly informative | For the polynomial regression terms, weakly informative priors were used to regularize higher-order effects while allowing the data to update the posterior. | μ=0, σ=0.5 |
| PC on X × Y | Normal(0,0.5) | Weakly informative | For the polynomial regression terms, weakly informative priors were used to regularize higher-order effects while allowing the data to update the posterior. | μ=0, σ=0.5 |
| PC on Y² | Normal(0,0.5) | Weakly informative | For the polynomial regression terms, weakly informative priors were used to regularize higher-order effects while allowing the data to update the posterior. | μ=0, σ=0.5 |
| PC on Mean Team Goal Commitment | Normal(0,0.5) | Weakly informative | For the polynomial regression terms, weakly informative priors were used to regularize higher-order effects while allowing the data to update the posterior. | μ=0, σ=0.5 |
| PC on Mean Team Goal Commitment of Others | Normal(0,0.5) | Weakly informative | For the polynomial regression terms, weakly informative priors were used to regularize higher-order effects while allowing the data to update the posterior. | μ=0, σ=0.5 |
| PC on Mean X² | Normal(0,0.5) | Weakly informative | For the polynomial regression terms, weakly informative priors were used to regularize higher-order effects while allowing the data to update the posterior. | μ=0, σ=0.5 |
| PC on Mean X × Y | Normal(0,0.5) | Weakly informative | For the polynomial regression terms, weakly informative priors were used to regularize higher-order effects while allowing the data to update the posterior. | μ=0, σ=0.5 |
| PC on Mean Y² | Normal(0,0.5) | Weakly informative | For the polynomial regression terms, weakly informative priors were used to regularize higher-order effects while allowing the data to update the posterior. | μ=0, σ=0.5 |
| PC on σ _Person:Intercept_ | Student-t(3,0,1) | Weakly informative | Based on the pilot data, the Student-t prior allows for heavier tails and accommodates heterogeneity among individuals. | ν=3, σ=1 |
| PC on σ _Team:Intercept_ | Student-t(3,0,0.6) | Moderately informative | Pilot ICC estimates revealed that there was less variability between teams than between individuals. Therefore, the prior reflects moderate team variance while avoiding extreme estimates. | ν=3, σ=0.6 |
| PC on σ _Team:Team Goal Commitment_ ^a^ (X) | Normal(0,0.5) | Weakly informative | Pilot data indicated minimal slope variation; prior constraints limit the parameter to a small but non-zero range, allowing the data to meaningfully update the posterior distribution. | μ=0, σ=0.5 |
| PC on σ _Team:Team Goal Commitment of Others_ ^a^ (Y) | Normal(0,0.5) | Weakly informative | Pilot data indicated minimal slope variation; prior constraints limit the parameter to a small but non-zero range, allowing the data to meaningfully update the posterior distribution. | μ=0, σ=0.5 |
| PC on σ _Team:X²_ | Normal(0,0.5) | Weakly informative | For the polynomial regression terms, weakly informative priors were used to regularize higher-order effects while allowing the data to update the posterior. | μ=0, σ=0.5 |
| PC on σ _Team:X × Y_ | Normal(0,0.5) | Weakly informative | For the polynomial regression terms, weakly informative priors were used to regularize higher-order effects while allowing the data to update the posterior. | μ=0, σ=0.5 |
| PC on σ _Team:Y²_ | Normal(0,0.5) | Weakly informative | For the polynomial regression terms, weakly informative priors were used to regularize higher-order effects while allowing the data to update the posterior. | μ=0, σ=0.5 |
| Team RE correlations | LKJ(4) | Regularizing | The LKJ prior was used to reduce extreme correlations among random effects, while allowing for correlations if the data supported them. | η=4 |
| Residual correlations | LKJ(4) | Regularizing | Residual correlations between outcomes were allowed while shrinking extreme dependencies estimates. | η=4 |
|  |  |  |  |  |

Default priors in brms were overridden by specified priors where indicated. **RC = relationship conflict**; **TC = task conflict**; **PC = process conflict**; RE = Random-Effects; LKJ = Lewandowski, Kurowicka, and Joe (initials of LKJ prior inventors). ^a^ **group-mean centered**.

1 References

Shah, P. P., Peterson, R. S., Jones, S. L., and Ferguson, A. J. (2021). Things are not always what they seem: The origins and evolution of intragroup conflict. *Adm. Sci. Q.* 66, 426–474. doi: 10.1177/0001839220965186.
